# Different signaling patterns contribute to loss of keratinocyte cohesion dependent on autoantibody profile in pemphigus

**DOI:** 10.1038/s41598-017-03697-7

**Published:** 2017-06-15

**Authors:** Elias Walter, Franziska Vielmuth, Lukas Rotkopf, Miklós Sárdy, Orsolya N. Horváth, Matthias Goebeler, Enno Schmidt, Rüdiger Eming, Michael Hertl, Volker Spindler, Jens Waschke

**Affiliations:** 1 Institute of Anatomy and Cell Biology, Ludwig-Maximilians-Universität München, Munich, 80336 Germany; 2 Department of Dermatology and Allergology, Ludwig-Maximilians-Universität München, Munich, 80336 Germany; 3 Department of Dermatology, Venerology and Allergology, University Hospital Würzburg, Würzburg, 97080 Germany; 4 Lübeck Institute of Experimental Dermatology (Lied), University of Lübeck, Lübeck, 23562 Germany; 5 Department of Dermatology and Allergology, Philipps-Universität Marburg, Marburg, 35037 Germany

## Abstract

Pemphigus is an autoimmune blistering skin disease caused primarily by autoantibodies against desmoglein (Dsg)1 and 3. Here, we characterized the mechanisms engaged by pemphigus IgG from patients with different clinical phenotypes and autoantibody profiles. All pemphigus vulgaris (PV) and pemphigus foliaceus (PF) IgG and AK23, a monoclonal mouse antibody against Dsg3, caused loss of cell cohesion, cytokeratin retraction and p38MAPK activation. Strong alterations in Dsg3 distribution were caused by mucosal (aDsg3 antibodies), mucocutaneous (aDsg1 + aDsg3) as well as atypical (aDsg3) PV-IgG. All PV-IgG fractions and AK23 compromised Dsg3 but not Dsg1 binding and enhanced Src activity. In contrast, rapid Ca^2+^ influx and Erk activation were induced by mucocutaneous PV-IgG and pemphigus foliaceus (PF) IgG (aDsg1) whereas cAMP was increased by mucosal and mucocutaneous PV-IgG only. Selective inhibition of p38MAPK, Src or PKC blocked loss of keratinocyte cohesion in response to all autoantibody fractions whereas Erk inhibition was protective against mucocutaneous PV-IgG and PF-IgG only. These results demonstrate that signaling patterns parallel the clinical phenotype as some mechanisms involved in loss of cell cohesion are caused by antibodies targeting Dsg3 whereas others correlate with autoantibodies against Dsg1. The concept of key desmosome regulators may explain observations from several experimental models of pemphigus.

## Introduction

Pemphigus is an autoimmune dermatosis characterized by blistering of the epidermis and/or mucous membranes^1^. Affected patients develop autoantibodies which bind to the cell-cell adhesion molecules desmoglein 1 (Dsg1) and/or Dsg3. These proteins belong to the cadherin superfamily of adhesion molecules and interact with corresponding molecules of the opposing cells extracellular domain. Intracellularly, they represent attachment zones for keratin filaments via the desmosomal plaque proteins desmoplakin (DP), plakoglobin (PG) and different plakophilin (PKP) isoforms^2^. Two main clinical variants of pemphigus exist, pemphigus vulgaris (PV) and pemphigus foliaceus (PF)^3^. PV has two main phenotypes: mucosal PV (m-PV), associated with autoantibodies against Dsg3 and exclusive effects on mucous membranes, and mucocutaneous PV (mc-PV), caused by autoantibodies targeting both Dsg3 and Dsg1 with additional blister formation in the suprabasal epidermis. In contrast, PF displays a phenotype restricted to blistering in the superficial epidermis with autoantibodies against Dsg1 only. Occasionally, the phenotype differs from this autoantibody pattern leading to atypical pemphigus variants (at-PV), i.e. an exclusive epidermal involvement in PV positive for anti-Dsg3 antibodies only or a combination of anti-Dsg3 and anti-Dsg1^4, 5^. On a molecular level, autoantibody binding results in disturbed desmosome turnover and depletion of desmoglein molecules as well as severe structural alterations of desmosomes such as the reduction of desmosome size and number and uncoupling of keratin filaments^6–8^. The mechanisms leading to these changes are only partially understood^9, 10^. After identification of desmosomal adhesion molecules as the primary antigens it was proposed that the autoantibodies sterically hinder the interaction of desmogleins, leading to loss of cell cohesion and consequently to blister formation^11, 12^. Indeed, atomic force microscopy (AFM) studies showed that PV autoantibody fractions (PV-IgG) containing anti-Dsg3 and anti-Dsg1 block the homophilic binding of Dsg3 but, similar to PF-IgG, do not alter Dsg1 interactions^13–15^. Alternatively, it was shown that modulation of signaling events prevents pemphigus skin blistering in a set of different models^16^. These pathways include, but are not limited to, Ca^2+^ influx^17^, protein kinase C (PKC)^18^, sarcoma-associated kinase (Src)^19, 20^ and p38 protein-activated kinase (p38MAPK) signaling^21^. Specifically for p38MAPK it was shown that pharmacologic inhibition protected against the pathogenic effects of PV-IgG under conditions in which the interaction of Dsg3 molecules was hindered^22^. It is possible that loss of Dsg3 interaction by anti-Dsg3 antibody binding in turn initiates specific signaling events^23^ and that both mechanisms are required for pathogenicity. Until now, comprehensive insight connecting the different signaling pathways with autoantibody profiles and clinical phenotypes is lacking. In this study, we thus applied IgG fractions from patients with typical PV and PF and one atypical case of PV and evaluated (i) the potency to induce pemphigus hallmarks such as Dsg3 depletion and keratin retraction, (ii) their impact on the activity of specific signaling events and (iii) the relevance of these signaling events for loss of cell cohesion. We found signaling patterns correlating with clinical phenotype and autoantibody profile and observed key mechanisms for desmosome regulation including p38MAPK-, PKC- and Src activation which may resolve the question why in several models of pemphigus inhibition of one single signaling pathway is protective.

## Results

### Pathogenic effects of pemphigus autoantibodies

**Figure 1:**
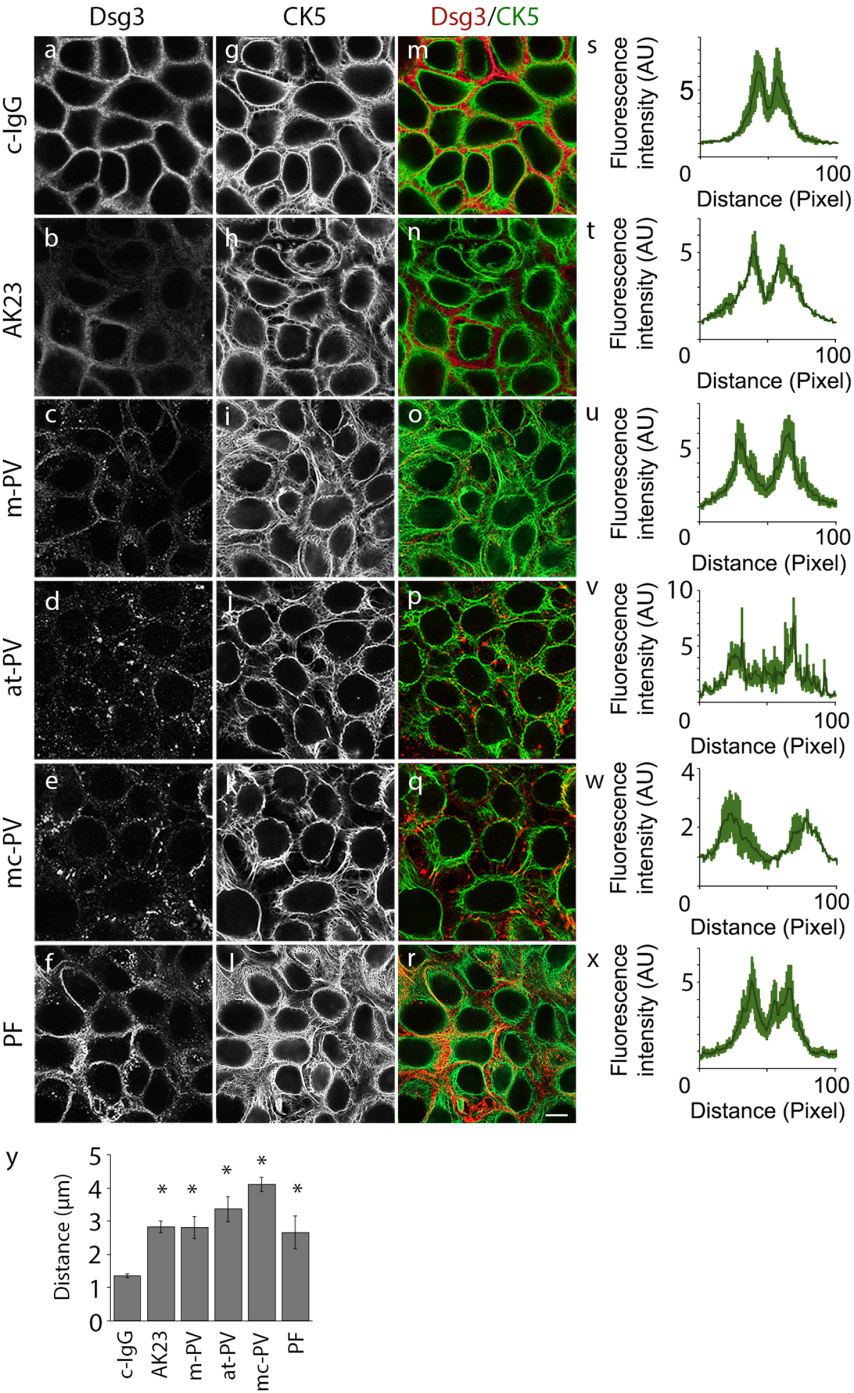
Structural changes in keratinocytes after 24 h of IgG treatment. HaCaT keratinocytes stably expressing CK5-YFP were exposed to different IgG fractions for 24 h. Images shown are representatives of four independent experiments. Dsg3 staining (**a**–**f**) and CK5 expression (**g**–**l**) after IgG treatment. (**m**–**r**) Merged images of Dsg3 staining and CK5-YFP. (**s**–**x**) CK5 fluorescence intensity of a 100 pixel long line placed centrally over the cell membrane (n = 4). (**y**) Analysis of the distance between fluorescence peaks shown in (**s**–**x**) in *μ*m (n = 4, *p ≤ 0.05 vs. c-IgG). Bar represents 10 *μ*m.

**Figure 2:**
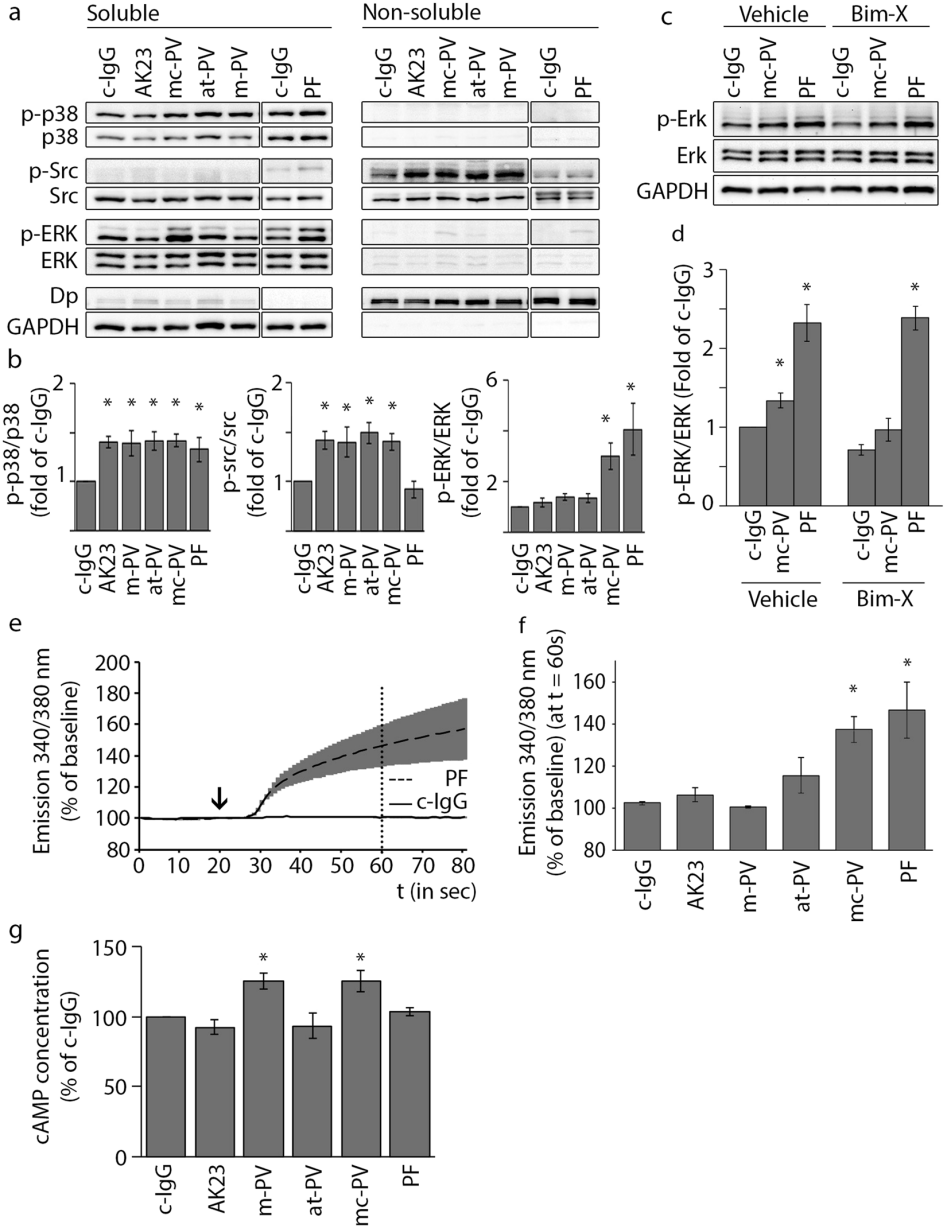
Signaling pathways activated by different IgG fractions. (**a**) Triton fractionation of HaCaT lysates into a TX-100 insoluble cytoskeletal and a TX-100 soluble non-cytoskeletal pool. Representative cropped Western blots of p38MAPK, src and Erk activity for each pool. (**b**) Densitometric analysis of signaling molecule phosphorylation normalized to the total amount in percent of control (n = 5–14, *p ≤ 0.05 vs. c-IgG). (**c**) Erk activation after inhibition of PKC by Bim-X and subsequent incubation of mc-PV and PF-IgG for 30 min. (**d**) Densitometric analysis of Erk phosphorylation corresponding to c (n = 4, *p ≤ 0.05 vs. respective c-IgG condition). Ratiometric fluorescent intensity measurement of Fura-2-AM at 340/380 nm normalized to baseline. (**e**) Graphs of c-IgG and PF-IgG to illustrate experimental setup. IgG incubation started at 20 s (arrow) and (**f**) concentrations were analyzed 60 s afterwards (dotted line) (n = 3, *p ≤ 0.05 vs. c-IgG). (**g**) Measurement of intracellular cAMP concentrations in keratinocytes after 6 h of IgG treatment (n = 4; *p ≤ 0.05 vs. c-IgG).

In this study, we compared autoantibody fractions from patients with m-PV, mc-PV, at-PV and PF (Table 1) with effects induced by the monoclonal autoantibody AK23 targeting Dsg3^24^ in HaCaT keratinocytes expressing both Dsg1 and Dsg3 (Supplementary Fig. S1). All autoantibody fractions recognized Dsg1 and/or Dsg3 in ELISA assays depending on their antibody profile (Table 1). In addition, under the reducing conditions of the SDS-PAGE, at-PV and, to a lesser extent, m-PV also recognize Dsg3 (Supplementary Fig. 1). In keratinocytes treated with IgG from a healthy volunteer, Cytokeratin (CK)5 intermediate filaments formed a dense meshwork closely apposed to Dsg3 distributed along cell junctions in desmosomal contacts (Fig. 1a and g). All autoantibody fractions induced detachment of filaments from cell junctions, a phenomenon referred to as keratin retraction and known to be a morphological hallmark of pemphigus (Fig. 1h–y). This was paralleled by loss of intercellular cohesion detected by dispase-based dissociation assays. In contrast, alterations in Dsg3 distribution were variable. Similar to previous studies, fragmentation of Dsg3 staining with formation of linear streaks was moderate in cells incubated with AK23 or PF-IgG (Fig. 2b and f) whereas all PV-IgG fractions caused strong clustering and depletion of Dsg3 (Fig. 1c–e) closely resembling effects in human epidermis of patients^13, 25, 26^.

**Table 1:** Antibody profiles of IgG-fractions determined by Dsg1 and Dsg3 ELISA and their clinical phenotypes.

	ELISA Score		Phenotype
	anti-Dsg3 U/ml	anti-Dsg1 U/ml	
c-IgG	—	—	healthy control
m-PV	2391.0	—	mucosal
m-PV2	162.9	—	mucosal
at-PV	153.4	—	atypical cutaneous
mc-PV	225.5	218.8	mucocutaneous
mc-PV2	172.5	154.8	mucocutaneous
PF	—	1645.0	cutaneous
PF2	—	266.4	cutaneous

### Signaling mechanisms triggered by pemphigus autoantibodies

Triton-X-100-mediated protein extraction was used to detect phosphorylation of p38MAPK, Src and Erk by Western blotting. Compared to control IgG, all autoantibodies increased p38MAPK activity, which was almost exclusively observed in the Triton-soluble fraction containing molecules not linked to the cytoskeleton (Fig. 2a and b). In contrast, Src activation only occurred in the desmosome-containing, non-soluble fraction, but was restricted to cells incubated with autoantibody fractions that contain aDsg3 and was absent in cells treated with PF-IgG lacking autoantibodies against Dsg3. In contrast, increased Erk phosphorylation was detected in the soluble fraction under conditions in which autoantibody fractions contained aDsg1 such as in mc-PV-IgG and PF-IgG compared to control. Because PKC activation was shown to be dependent on Ca^2+^ influx and we previously used Erk phosphorylation as a read-out for PKC activation^17, 18, 27^, we studied whether Erk activation was dependent on PKC activity (Fig. 2c and d). However, in the Triton-soluble fraction the PKC inhibitor Bim-X alone reduced both baseline Erk activity and Erk activation in response to mc-PV-IgG but not PF-IgG indicating that Erk activation was not primarily affected downstream of PKC. Next, we measured intracellular Ca^2+^ levels via the fluorescence ratio of FURA-2-AM emission (Fig. 2e and f). In line with the data aforementioned, significant Ca^2+^ increase was detectable after incubation with mc-PV-IgG and PF-IgG only. These data indicate that p38MAPK and Src can be activated by antibodies targeting Dsg3 whereas the Ca^2+^/PKC pathway as well as Erk is activated by IgG fractions containing aDsg1 only. These results were confirmed in normal human epithelial keratinocytes (NHEK) (Supplementary Fig. 2). Furthermore, we applied one additional IgG fraction for each phenotype yielding a similar outcome in regard to p38 and Erk activity as well as Ca^2+^ influx (Supplementary Fig. 3). Finally, since previously reports showed that intracellular cAMP levels can be augmented by PV-IgG^28^ we measured cAMP by ELISA under the conditions used for the present study (Fig. 2g). Increased cAMP was detected in response to m-PV-IgG and mc-PV-IgG selectively.

### Effects of autoantibodies on Dsg3 and Dsg1 interaction measured by AFM

**Figure 3:**
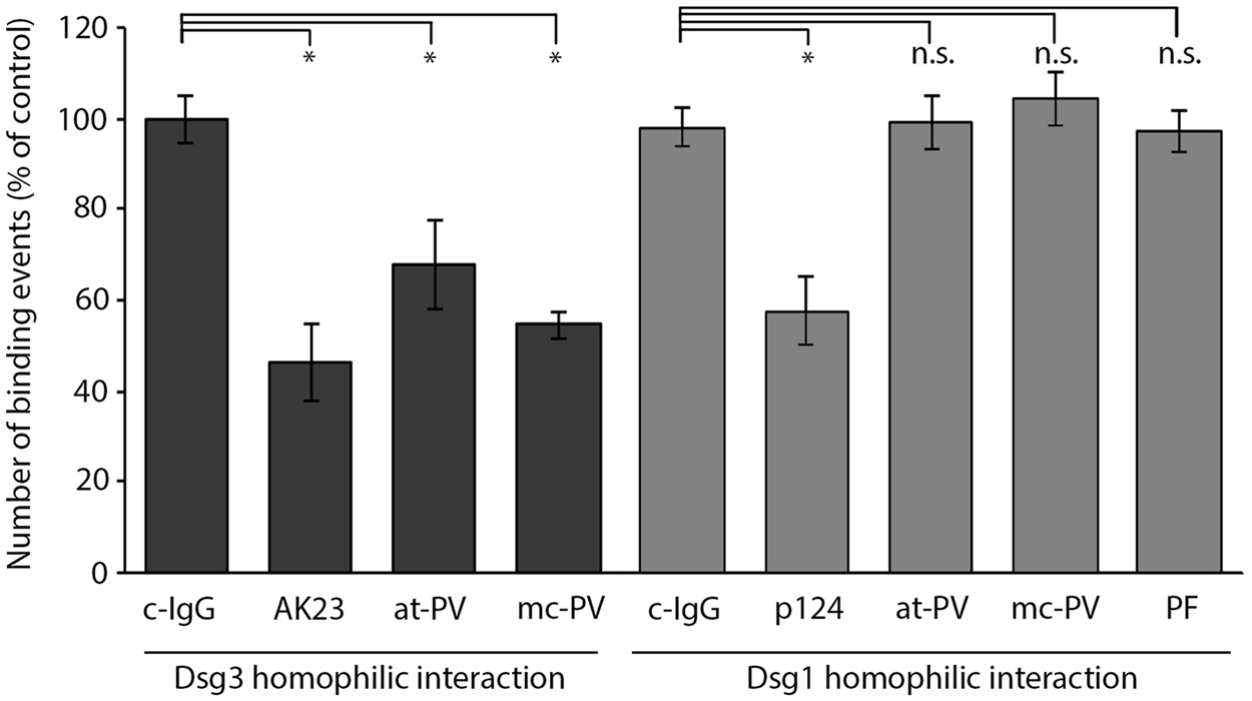
Inhibition of Dsg3 but not Dsg1 homophilic interactions by pemphigus autoantibodies. Cell free atomic force microscopy measurements on a coated mica sheet with an AFM tip functionalized with either recombinant Dsg1-Fc or Dsg3-Fc respectively. A grid with 10 × 10 points (2.5 × 2.5 *μ*m) was measured 5 times followed by incubation with IgG-fractions, AK23 or the mouse monoclonal Dsg1 antibody p124. The identical area was scanned again and normalized to the respective measurement before incubation (n = 3–5, *p ≤ 0.05 vs. c-IgG).

PV-IgG, but not PF-IgG, have been shown to interfere with interaction of Dsg3 but not Dsg1 on a single molecule level^13–15^. In line with this, in this study AK23, at-PV-IgG and mc-PV-IgG reduced Dsg3 binding as revealed by AFM (Fig. 3). In contrast, reduced number of Dsg1 binding events was observed by incubation with the commercially available inhibitory antibody p124 targeting Dsg1, but not with at-PV-IgG, mc-PV-IgG or PF-IgG although the latter two contained aDsg1. These data show that direct inhibition of Dsg3 binding correlated with the occurrence of aDsg3 and thus Src activation, whereas IgG fractions containing aDsg1 had no effects on Dsg1 interactions but nevertheless induced Ca^2+^/PKC and Erk-activation.

### Relevance of signaling pathways stimulated by autoantibodies for keratinocyte cohesion

**Figure 4:**
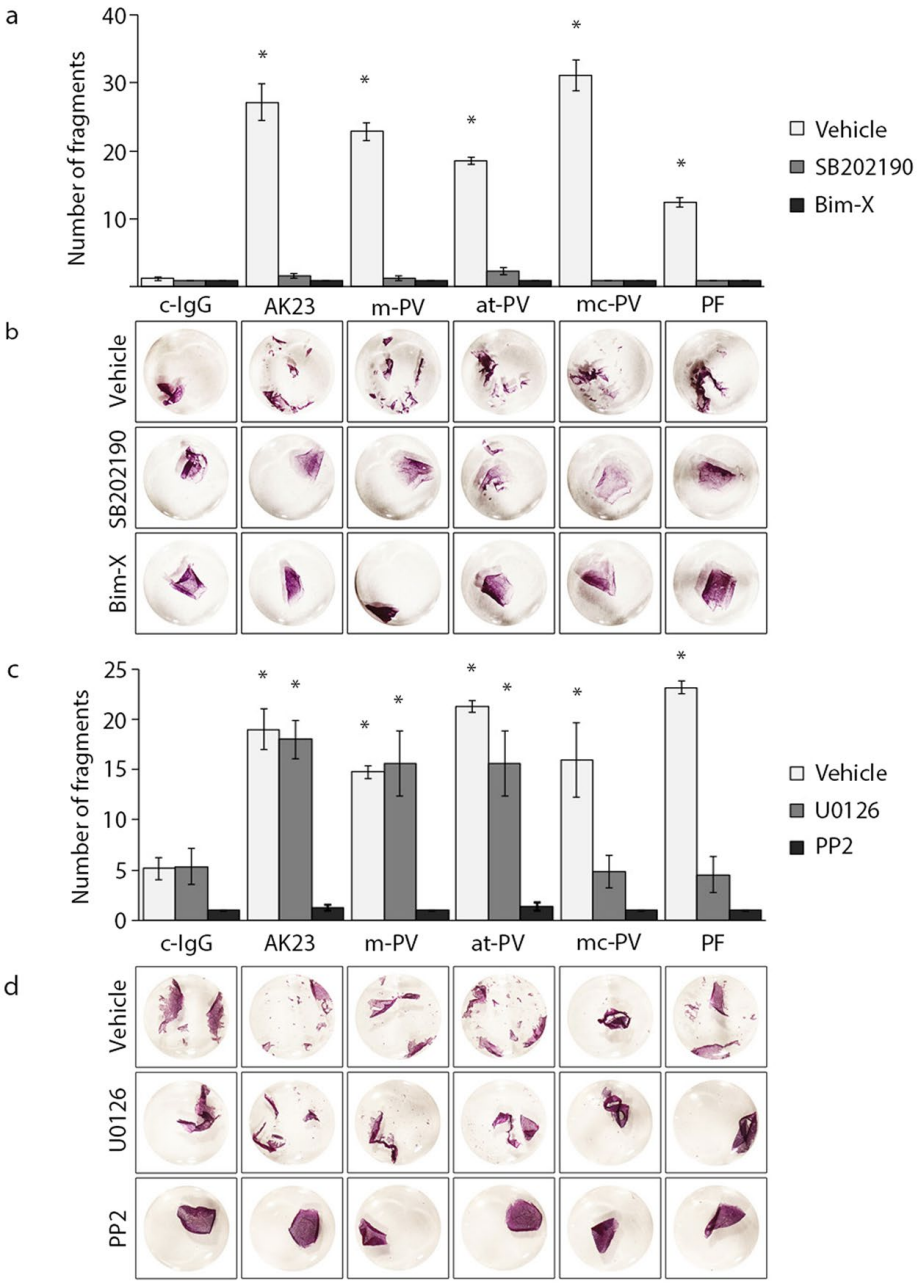
Adhesion measurements with modulation of signaling molecules. Dispase-based dissociation assays in HaCaT keratinocytes after applying the specific inhibitors SB202190 (p38MAPK), Bim-X (PKC), U0126 (Erk) or PP2 (Src) for 1 h, followed by either (**a**) 24 h incubation of IgG fractions or (**c**) 2 h incubation of IgG fractions (n = 4; *p ≤ 0.05 vs. c-IgG with the respective mediator). (**b**,**d**) Representative images of monolayers incubated with 10 *μ*m MTT to visualize cell fragments.

Finally, we addressed the relevance of signaling events triggered by autoantibodies for intercellular cohesion of keratinocytes. The *in vivo* relevance of p38MAPK, PKC and tyrosine kinases including Src for pemphigus pathogenesis has been shown before^21, 29–31^. Similar to most studies in the past, we incubated inhibitors for p38MAPK (SB202190) and PKC (Bim-X) with autoantibody fractions for 24 h (Fig. 4a). Loss of keratinocyte cohesion induced by all antibody fractions was abolished by SB202190 and Bim-X. Because inhibition of Erk for 24 h using U0126 was toxic, we incubated autoantibodies with U0126 or with the Src inhibitor PP2 for 2 h only, similar to a recent report on p38MAPK^22^. In contrast to PP2, which was protective under all conditions, U0126 reduced effects of mc-PV-IgG and PF-IgG only, i.e. the autoantibody fractions containing aDsg1 and leading to Erk activation (Fig. 4b).

## Discussion

For the first time we investigated under strictly the same settings whether signaling pathways activated by pemphigus IgG correlate with autoantibody profile and thus with clinical phenotype. Previously, it was shown that p38MAPK and RhoA signaling were modulated by both PV-IgG and PF-IgG, suggesting that autoantibodies may have similar effects in signaling^21, 32^. In contrast, we observed signaling patterns different for mucosal, atypical and mucocutaneous PV-IgG as well as for PF-IgG, which only partially paralleled the effects on Dsg3 interaction, in AFM experiments. The second important outcome of this study is that the potency of signaling pathway modulation to protect against loss of keratinocyte cohesion varies for different signaling mechanisms. From the latter observation, we conclude that the relevance of signaling mechanisms in the regulation of desmosomal binding is different and propose that some pathways including Src and PKC may have the role of key desmosome regulators under basal conditions whereas others are autoantibody-specific.

**Figure 5:**
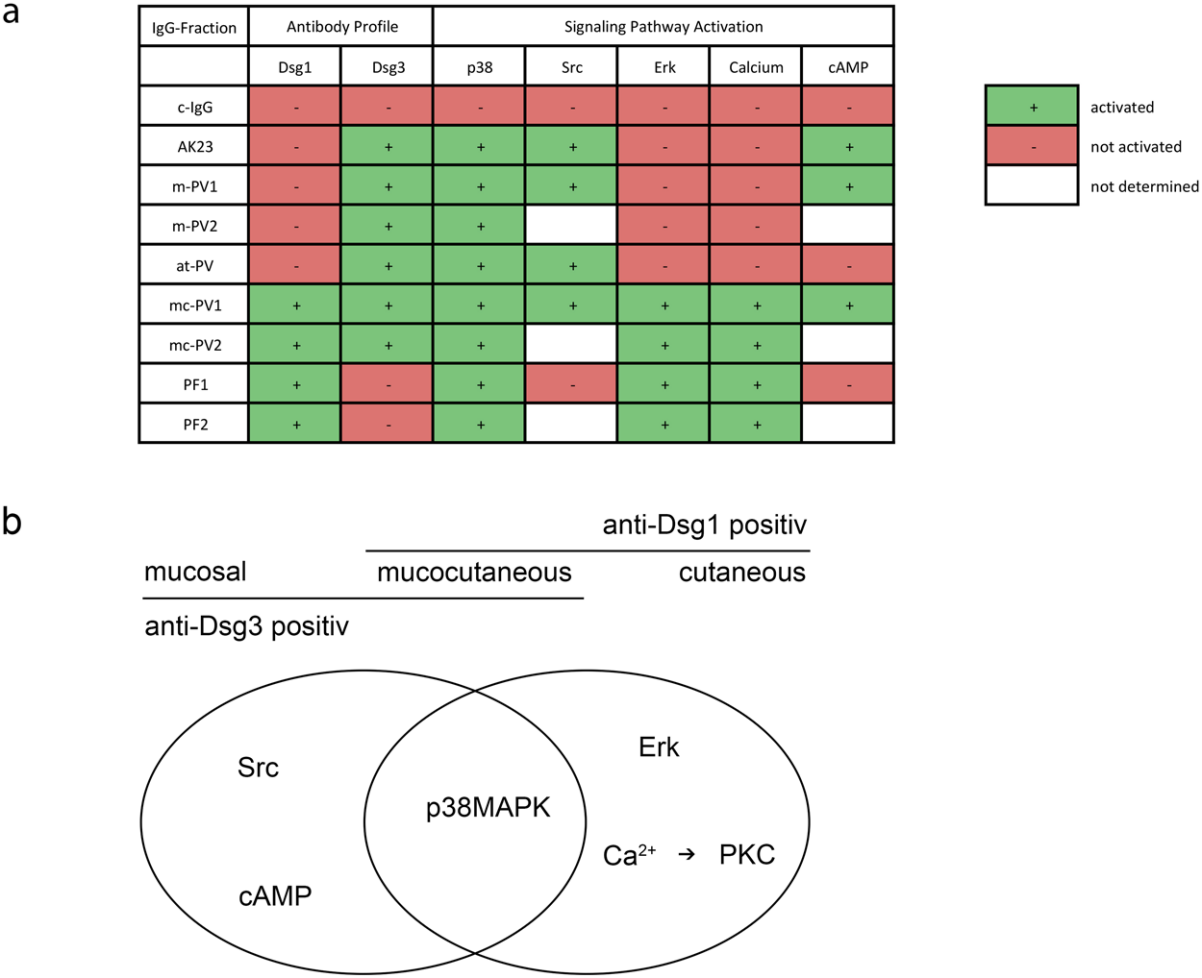
Signaling patterns associated with autoantibody profiles. Summary (**a**) and schematic (**b**) representation of signaling patterns associated with different autoantibody fractions and their respective clinical phenotypes.

The signaling patterns identified here appear to be complex. Activation of p38MAPK was found under all pathological conditions which is in line with all publications where a role of this pathway in pemphigus pathogenesis has been reported downstream of PV-IgG, PF-IgG and AK23^16, 19, 23, 25, 33–41^. This indicates that antibodies against Dsg3 as well as autoantibody fractions containing aDsg1 but not aDsg3 can activate p38MAPK signaling (Fig. 5). In contrast, Src appears to be activated by aDsg3 specifically because both the monoclonal antibody AK23 and PV-IgG but not PF-IgG stimulated this pathway as shown here. On the other hand, Ca^2+^ and Erk signaling pathways were restricted to IgG fractions containing aDsg1 only. Importantly, we cannot definitely conclude whether autoantibodies directed against Dsg1 were primarily responsible for signaling because additional antibodies present in the polyclonal IgG fractions may also be involved^42^. The identification of signaling pathways activated by specific desmosomal and non-desmosomal autoantibodies should be a primary focus for future studies. The data presented here suggest that autoantibodies targeting Dsg3 cause direct inhibition of Dsg3 binding as well as activation of some pathways such as p38MAPK and Src which contributes to keratin retraction and loss of keratinocyte cohesion at least under mechanical stress in the dissociation assay. Moreover, it is possible that p38MAPK and Src are relevant for Dsg3 clustering as proposed before^25, 33, 43^ although the role of this process leading to loss of cell cohesion is not clear since it was detected in unaffected skin of m-PV and mc-PV patients as well^26^. Thus, it can be speculated that p38MAPK- and Src-mediated loss of keratinocyte adhesion may be sufficient to cause mucosal PV but not blistering in human skin where usually acantholysis does not occur when antibodies targeting Dsg3 but not Dsg1 are present^44, 45^. Rather, Ca^2+^ and Erk signaling may be additionally required for epidermal blistering. However, since pemphigus pathogenesis is complex and autoantibodies against other antigens can be present as well^10, 42, 46, 47^, we do not expect that the signaling patterns are similar in each patient. For cAMP signaling, which was proposed to be a salvage pathway in PV by limiting p38MAPK activation and Dsg3 depletion^28^, we observed that levels of cAMP were elevated in response to m-PV-IgG and mc-PV-IgG only but not to AK23, at-PV-IgG or PF-IgG. This may indicate that some but not all antibodies targeting Dsg3 may activate cAMP signaling or that other autoantibodies are responsible. However, it is possible that the capability of autoantibodies to stimulate cAMP signaling may modulate the clinical phenotype as well because it could contribute to the fact that in m-PV skin blistering is absent but occurred in the patient with at-PV-IgG in our study in the absence of aDsg1. Accordingly, failure to induce this protective pathway may enhance the pathogenicity of AK23, which can cause epidermal blistering in mice^23, 24, 40^.

We found that selective inhibition not only of p38MAPK but also of Src and PKC was effective to completely abrogate loss of cell cohesion in response to PV-IgG and PF-IgG. This was surprising because PKC was activated by mc-PV-IgG and PF-IgG only whereas Src activation was confined to PV-IgG and AK23 treatment. This demonstrates that inhibition of some signaling molecules is protective although the activity state of the molecule is not altered by autoantibody incubation. One may conclude that Src and PKC are in general critical for desmosome function and thus may represent key regulators of desmosomal adhesion, e.g. by induction of hyperadhesion as outlined below. The efficacy of p38MAPK inhibition to protect against loss of keratinocyte cohesion as observed here is well established in response to pemphigus autoantibodies or depletion of Dsg3 and plakoglobin^9, 21, 34, 37, 43, 48, 49^. The underlying mechanisms may involve keratin retraction, depletion of Dsg3 from junctions as well as interference with formation of new desmosomes^16, 27, 33, 34^. For Src activation in response to PV-IgG, phosphorylation of PKP3 and detachment from Dsg3 may destabilize cell cohesion^19, 20, 50^. On the other hand, PKC was demonstrated to regulate keratin filament anchorage to DP and thereby sequestration of PKC to keratin filaments^51–53^. Moreover, inhibition of PKC-mediated DP phosphorylation as well as PKP1 overexpression rendered desmosomes hyperadhesive, i.e. Ca^2+^-independent^51, 52, 54^. Since PKC in this scenario was shown to be regulated by PKP3^55^, a crosstalk with Src signaling is conceivable. For Erk, which has also previously been reported to be activated in response to PV-IgG^56^, the situation appears to be different because Erk was activated when aDsg1 were present and contributed to loss of cell cohesion under this condition only. In contrast, inhibition of Erk did not prevent loss of cell cohesion induced by autoantibodies containing aDsg3 but not aDsg1. Future studies are required to unravel mechanisms by which Erk regulates desmosomal binding and to elucidate why Erk is not of equal importance for keratinocyte cohesion as p38MAPK, Src and PKC. The concept of central desmosomal regulators may explain why in many experimental models for pemphigus modulation of a single signaling mechanism was found to be protective. Taken together, our study enhances the understanding on pemphigus pathogenesis because it proposes that a pattern of signaling events, which are of different importance for regulation of desmosomal adhesion, may define the clinical phenotype of pemphigus.

## Methods

### Cell culture and test reagents

The human immortalized keratinocyte cell line HaCaT^57^ was cultured in Dulbecco’s Modified Eagle Medium containing 10% FCS (Biochrom, Berlin, Germany), 50 U/ml penicillin and 50 g/ml streptomycin (both AppliChem, Darmstadt, Germany) in a humidified atmosphere of 5% CO_2_ at 37 °C HaCaT cells stably transfected with keratin5-YFP were used for immunostaining experiments (kind gift of Reinhard Windoffer and Nicole Schwarz, Institute of Molecular and Cellular Anatomy, RWTH Aachen University^58^). The cells were used 1d after confluency and medium was routinely changed the evening before experimental procedure. Adult Normal Human Epithelial Keratinocytes (NHEK) were purchased and cultured in Keratinocyte Growth Medium 2 (both Promocell, Heidelberg, Germany) in low calcium conditions (0.06 mM) until confluency and eventually switched to 1.2 mM calcium for 24 h. Experiments were performed in between passages 3 and 6.

### Pemphigus sera and IgG purification

The pathogenic monoclonal antibody AK23 against the EC1 domain of Dsg3 was purchased (Biozol, Eching, Germany) and used at a concentration of 75 g/ml. To inhibit homophilic interactions of Dsg1, the monoclonal antibody Dsg1 p124 (Progen, Heidelberg, Germany) directed against the extracellular domain of Dsg1 was used in a 1:20 dilution. For purification of sera, patients gave written consent for research use and institutional approval of the ethics committee was given. The phenotype was characterized in the process of disease diagnosis via clinical and histological evaluation and the antibody profile was determined by Dsg1 and Dsg3 ELISA. IgG fractions were purified using protein A affinity chromatography as described previously^15^. Briefly, antibodies from patient sera were immobilized to protein A agarose (ThermoFisher, Waltham, USA) in purification columns for 2 h at room temperature. After washing with phosphate-buffered saline (PBS), the antibody fractions were eluted by sodium citrate buffer (20 mM, pH 2.4), neutralized with Na_2_CO_3_ and concentrated in a filter unit (Amicon Ultra–4, 100k; Merck Millipore, Darmstadt, Germany) at 19000 g for 20 min. Afterwards, the IgG-fractions were retrieved with PBS and tested for functionality in a dispase-based dissociation assay.

### Immunostaining

Cells were washed with PBS and fixed with 2% formalin (freshly prepared from paraformaldehyde) in PBS for 10 min. Afterwards they were permeabilized with 1% Triton X-100 in PBS for 5 min. Subsequently, cells were blocked by 3% bovine serum albumin and 1% normal goat serum in PBS for 60 min and incubated with anti-Dsg3 mAb (clone 5G11, Invitrogen, Carlsbad, CA) at 4 °C overnight. The Cy3-labeled secondary goat anti-rabbit antibody (Dianova, Hamburg, Germany) was incubated for 1 h at room temperature and samples were mounted with n-propyl gallate as an antifade compound on glass slides. Images were acquired with a 63x NA 1.4 PL APO objective on a SP5.II confocal microscope (both Leica, Mannheim, Germany).

### Triton X-100 protein fractionation, electrophoresis and Western blotting

After washing with ice-cold PBS, cell lysates were retrieved by incubation in extraction buffer (0.5% Triton X-100, 50 mmol/L MES, 25 mmol/L EGTA, 5 mmol/L MgCl_2_) for 10 min on ice containing a mixture of 0,1% leupeptin, aprotinin and pepstatin as well as 1% phenylmethylsulfonylfluorid under gentle shaking. Afterwards cells were scraped and centrifuged at 19000 g for 10 min at 4 °C to separate the triton-soluble, non cytoskeletal-bound fraction from the cytoskeletal bound insoluble fraction (below named pellet). The pellet was suspended in SDS-lysis buffer (25 mmol/l HEPES, 2 mmol/l EDTA, 25 mmol/l NaF and 1% SDS, pH 7,4) and sonicated. Eventually, the protein amount was measured using the BCA method (Thermo Fisher Scientific, Schwerte, Germany). Both fractions were mixed with laemmli buffer containing 50 mM dithiothreitol. Electrophoresis and wet blotting were performed according to standard procedures. Membranes were incubated at 4 °C overnight with the following primary antibodies in 5% bovine serum albumin (BSA) in tris-buffered-saline with 0.05% tween (TBS-T): phospho-p38 MAPK pAB (Thr180/Tyr182) (Cell Signaling Technologies, Cambridge, United Kingdom), p38 MAPK pAB (Cell Signaling Technologies), p-Erk mAB (E-4) (Santa Cruz, Heidelberg, Germany), p44/42 MAPK pAB (Erk1/2) (Cell Signaling Technologies), p-src pAB (Tyr416) (Cell Signaling Technologies), Src pAB (Cell Signaling Technologies), Dsg3 mAB (5H10) (Novus Biological, Littleton, USA), Dsg1 mAB (p124) (Progen), GAPDH mAB (Santa Cruz), and Desmoplakin I/II pAB (H-300) (Santa Cruz). Secondary antibodies were either a HRP-coupled goat anti-mouse, a HRP-coupled goat anti-rabbit or a HRP-coupled goat anti-human mAB (Dianova, Hamburg, Germany). Chemiluminescence was detected with the ECL system (GE Healthcare, Munich, Germany). Western Blots were cropped at the appropriate kDa size. Full-length versions can be found in Supplementary Fig. 4.

### Measurements of cellular cAMP levels

Cellular cAMP levels were determined by cAMP enzyme linked immunosorbent assay (CA-200) (Sigma-Aldrich, St. Louis, MO). After 6 h of IgG incubation cells were washed and lysed by 0.1 M HCl. The assay was further performed according to manufacturer’s instructions.

### Ca^2+^ concentration measurements

Cells were washed with measurement buffer (140 mM NaCl, 3.6 mM KCl, 2.6 mM CaCl_2_(H_2_O)_2_, 0.5 mM NaH_2_PO_4_(H_2_O)_2_, 2 mM NaHCO_3_, HEPES and 5 mM D + Glucose) prior to experiment performance. A mixture of 0.4% Fura-2-AM (Molecular Probes, Thermo Fisher) and 0.4% Pluronic F-127 (Life Technologies, Thermo Fisher) diluted in measurement buffer were afterwards incubated for 20 min at 37 °C. Cells were subsequently incubated in fresh measurement buffer for another 15 min before analysis was started. The experiments were performed with an inverted microscope (Carl Zeiss Microscopy) equipped with a polychrome V light source (TILL Photonics, Martinsried, Germany) a CoolSNAP-Hq2 digital camera (Photometrics) and a Fura-2 filter set (AHF Analysetechnik, Tuebingen, Germany).

### Atomic force microscopy (AFM)

Measurements were performed as previously described by our group^41^ under cell-free conditions using a NanoWizard® 3 AFM (JPK-Instruments, Berlin, Germany). Cleaved mica sheets (SPI-Supplies, West Chester, USA) and pyramidal-shaped D-Tips of Si_3_N_4_ MLCT cantilevers with a nominal spring constant of 0.03 N/m and a 20 nm tip radius (Bruker, Mannheim, Germany) were coated with a flexible heterobifunctional acetal-polyethylenglycol linker (PEG, Hermann Gruber Lab, Institute of Biophysics, Linz, Austria) and in turn functionalized with purified Dsg1-Fc and Dsg3-Fc (both 0.15 mg/ml). In force spectroscopy mode (FS-mode), the cantilever was repeatedly lowered onto and retracted from the mica sheet with a pulling speed of 1 *μ*m/s, a z-length of 0.3 *μ*m and a resting contact time of 0.1 s. An area of 25 × 25 *μ*m was selected and 500 measurement positions were probed in a grid of 10 × 10 readings for five times in HBSS buffer. The same area on the mica sheet was scanned for binding events with the same specifications before and after addition of IgG-fractions for 1 h. By calibration using the thermal-noise method^59^, deflections of the cantilever were converted into forces and plotted as force-distance curves. These force curves were evaluated for unbinding events, indicating interactions between tip and mica-attached Dsg molecules. The number of unbinding events per total number of approach/retract cycles was determined as binding frequency. Binding frequency after addition of pemphigus autoantibodies was normalized to the value before measurement at the same area.

### Dispase-based dissociation assay

The cell monolayer was detached from the well bottom by a 20 min incubation step with Dispase-II (Sigma-Aldrich) after thorough washing with HBSS (Sigma Aldrich). Afterwards, Dispase-II solution was substituted by HBSS and mechanical stress was applied by shearing the monolayer 10 times with an electrical 1 ml pipette. The cell fragments, counted under a binocular microscope (Leica), are a measure for loss of cell cohesion. Cells were incubated for 10 min in 10 *μ*m thiazolyl blue tetrazolium bromide (MTT) (Sigma Aldrich) for better visibility.

### Data analysis and statistics

Images were processed using Photoshop CC (Adobe Systems, San Jose, CA). Densitometric measurements were performed using the gel analysis function in ImageJ (Wayne Rasband, www.nih.gov). Data were analyzed in Excel (Microsoft, Redmond, WA). Statistical analysis was performed using one way ANOVA followed by Dunn-Šidák correction using Graphpad Prism (Graphpad Software, LaJolla, CA). Significance was presumed with p ≤ 0.05. Data are shown as mean ± SEM. Each n represents one independent experiment.

## Electronic supplementary material

Supplementary Material
