# Supplementary material for: Different signaling patterns contribute to loss of keratinocyte cohesion dependent on autoantibody profile in pemphigus
Source: Sci Rep. 2017 Jun 15;7:3579. doi: 10.1038/s41598-017-03697-7 (PMC5472593; doi:10.1038/s41598-017-03697-7)
Supplement: Supplementary file 1 — Supplementary Material [file 41598_2017_3697_MOESM1_ESM.pdf]

Different signaling patterns contribute to loss of keratinocyte cohesion dependent on autoantibody profile in pemphigus

Elias Walter[1], Franziska Vielmuth[1], Lukas Rotkopf[1], Miklós Sárdy[2], Orsolya N. Horváth[2], Matthias Goebeler[3], Enno Schmidt[4], Rüdiger Eming[5], Michael Hertl[5], Volker Spindler[1,*], and Jens Waschke[1,*]

[1]Institute of Anatomy and Cell Biology, Ludwig-Maximilians-Universität München, Munich, 80336, Germany
[2]Department of Dermatology and Allergology, Ludwig-Maximilians-Universität München, Munich, 80336, Germany
[3]Department of Dermatology, Venerology and Allergology, University Hospital Würzburg, Würzburg, 97080, Germany
[4]Lübeck Institute of Experimental Dermatology (Lied), University of Lübeck, Lübeck, 23562, Germany
[5]Department of Dermatology and Allergology, Philipps-Universität Marburg, Marburg, 35037, Germany
*Volker.Spindler@med.uni-muenchen.de
*Jens.Waschke@med.uni-muenchen.de

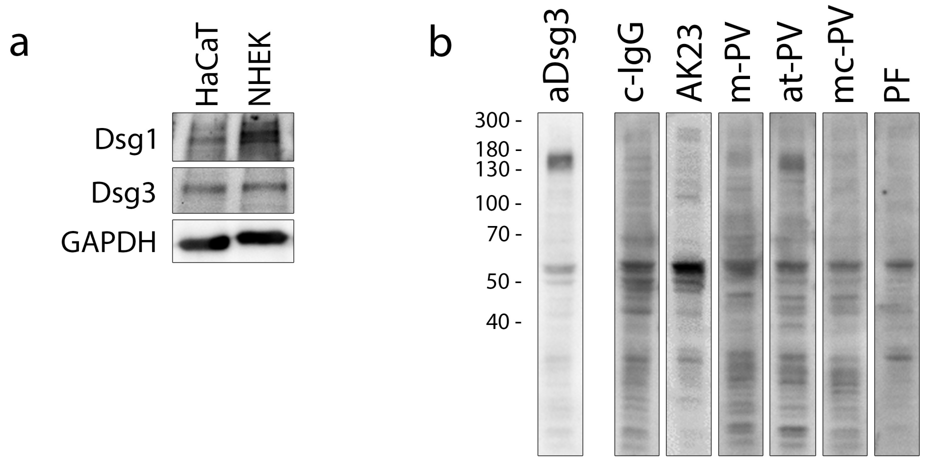

Supplementary Figure 1

Characterisation of Desmoglein 1 and 3 expression and PV-IgG Profile
(a) Representative Western Blot of HaCaT and NHEK cell lysates showing Desmoglein 1 and Desmoglein 3 expression (n=3). Representative Western Blot of HaCaT cell lysates incubated with the IgG-fraction as a primary antibody (n=3).

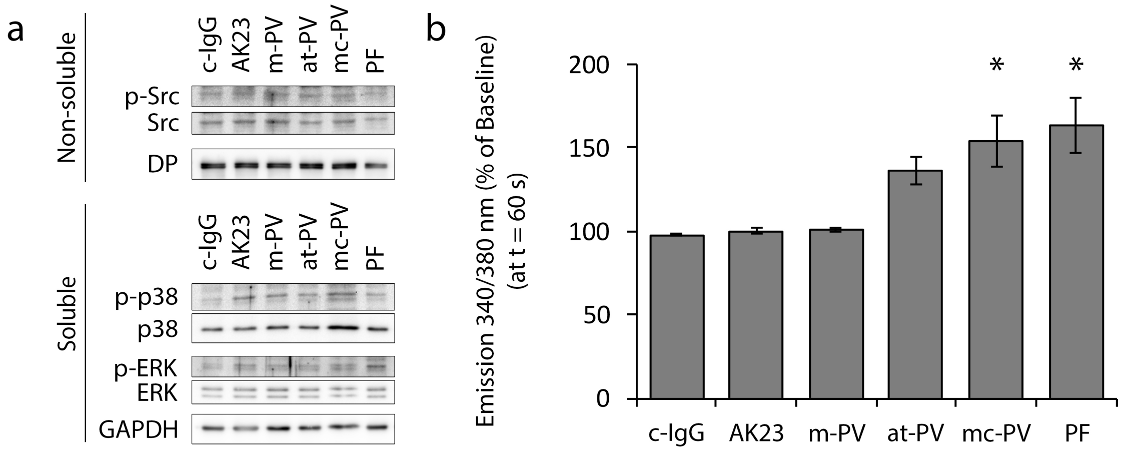

Supplementary Figure 2

Signaling pathway activation in normal human epithelial keratinocytes
Triton fractionation after IgG incubation in NHEKs. (a) Representative Western Blot for Src, p38 and ERK activation in the respective pool (n=3). (b) Calcium influx measured by ratiometric fluorescent intensity of 340/380 nm after IgG incubation in NHEKs (n=3; * p≤0.05 vs. c-IgG).

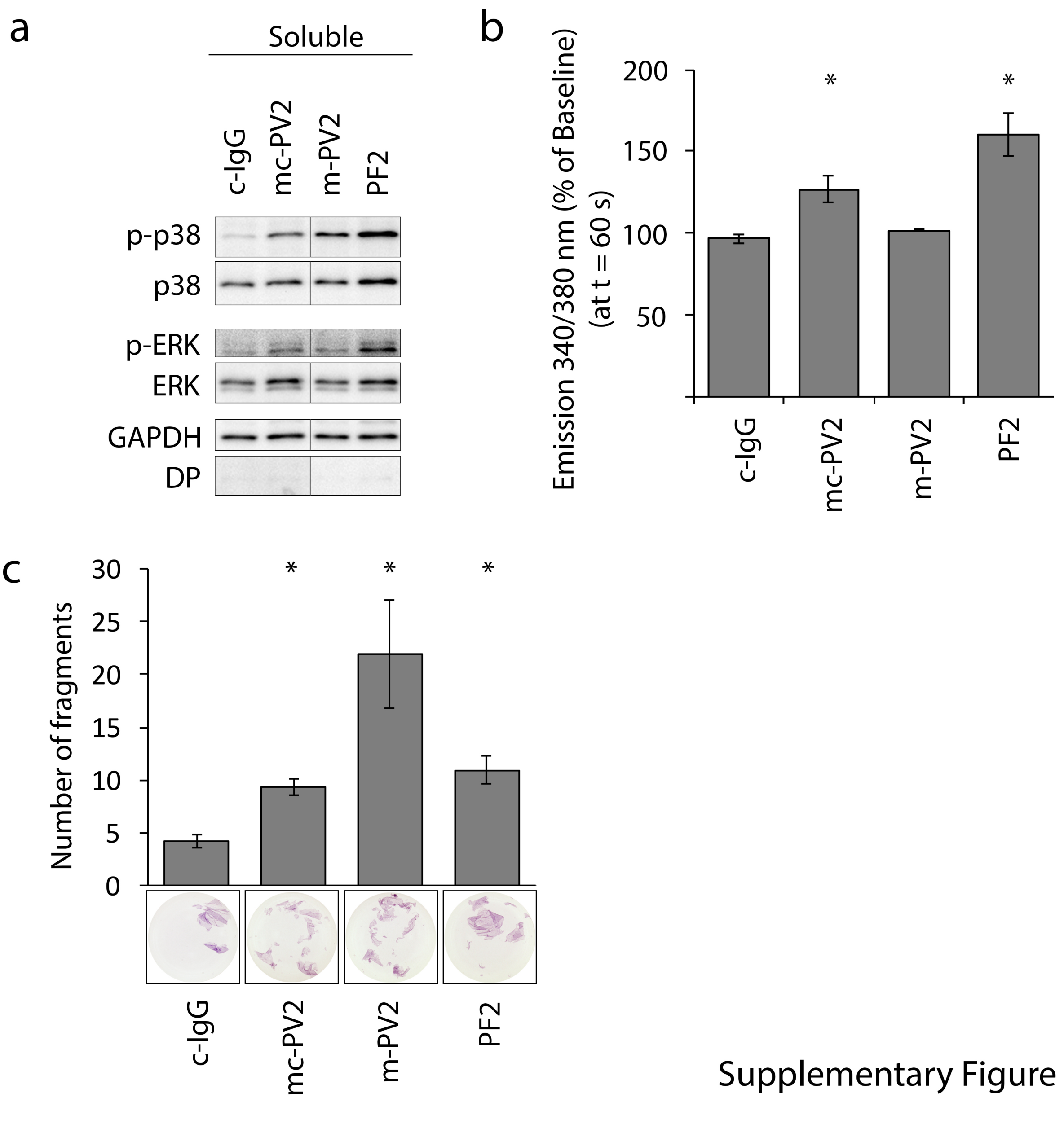

Supplementary Figure 3

Signaling pathway activation of additional IgG fractions

Experiments were performed in HaCaT keratinocytes. Triton fractionation after IgG incubation for 30 min. (a) Representative Western Blot for p38 and ERK activation in the soluble fraction (n=3). (b) Calcium influx measured by ratiometric fluorescent intensity of 340/380 nm after IgG incubation (n=3; * p≤0.05 vs. c-IgG). Fragments in a dissociation assay after 24 h of IgG incubation (n=3; * p≤0.05 vs. c-IgG).

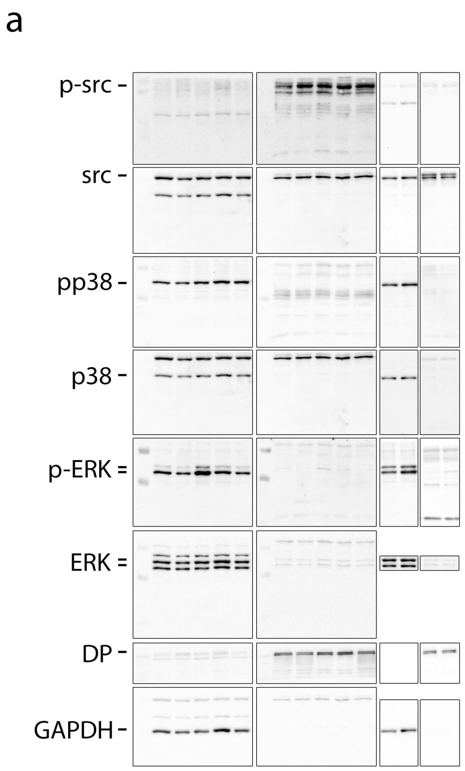

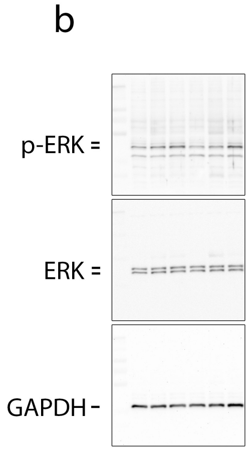

Supplementary Figure 4

Full-lenght Western Blots

Full-lenght Western Blots of cut-out sections in Figure 2a (a) and 2c (b). Protein of interest and sections are marked by a line.
